# 2224. A Novel Pediatric Antimicrobial Stewardship Program Intervention Leads to Decreased Use of Piperacillin-tazobactam in the Neonatal Intensive Care Unit at an Academic Institution

**DOI:** 10.1093/ofid/ofad500.1846

**Published:** 2023-11-27

**Authors:** Patricia Pichilingue-Reto, Kelsey Trimble, Marissa Johnston, Maria G Dominguez-Garcia, Katherine C Hill

**Affiliations:** Louisiana State University Health Sciences Center Shreveport, Shreveport, Louisiana; Ochsner LSU Health Shreveport, Shreveport, Louisiana; Ochsner LSU Health Shreveport, Shreveport, Louisiana; Louisiana State University Health Sciences Center Shreveport, Shreveport, Louisiana; University of Texas Southwestern Medical Center, Dallas, Texas

## Abstract

**Background:**

Antimicrobial stewardship programs (ASP) were created to improve antimicrobial use without compromising patient outcomes. Nationwide, Pediatric ASPs have shown a very rapid growth during the past decade. Inpatient stewardship can present some challenges, particularly with special patient populations like newborns and premature infants.

**Methods:**

A new Pediatric Antimicrobial Stewardship committee was established in April 2022 at Ochsner LSU Health Shreveport- St. Mary’s Hospital. The first process measurement was a medication usage evaluation (MUE) report of piperacillin-tazobactam from January 31st, 2020, to September 7th, 2021, in the Neonatal Intensive Care Unit (NICU) for early and late onset sepsis. Neonatologists received education about the recommended antibiotics for these indications. The stewardship committee obtained a report one year later to evaluate the impact of the intervention. Slicer dicer, a data extraction tool from EPIC Electronic Health Record (EHR) system was used to determine how many NICU patients received piperacillin-tazobactam.

**Results:**

From January 31st, 2020, to September 7th, 2021, the charts of 64 neonatal patients, who were prescribed piperacillin-tazobactam, were reviewed. Out of these 64 patients, 46 (72%) were 4-28 days old when the antibiotic was received, 33 (72%) received piperacillin-tazobactam and vancomycin and 13 received additional antibiotics. Indications for the 46 patients included: Possible sepsis (15%), confirmed sepsis (6.5%), possible necrotizing enterocolitis (NEC) (2.2%) and confirmed NEC (10.9%). The report demonstrated a possible overuse of piperacillin-tazobactam for early and late onset sepsis, without the diagnosis of NEC. We reported this antibiotic’s usage in NICU patients during the years 2020, 2021 and 2022 (Figure 1).

**Figure 1:**
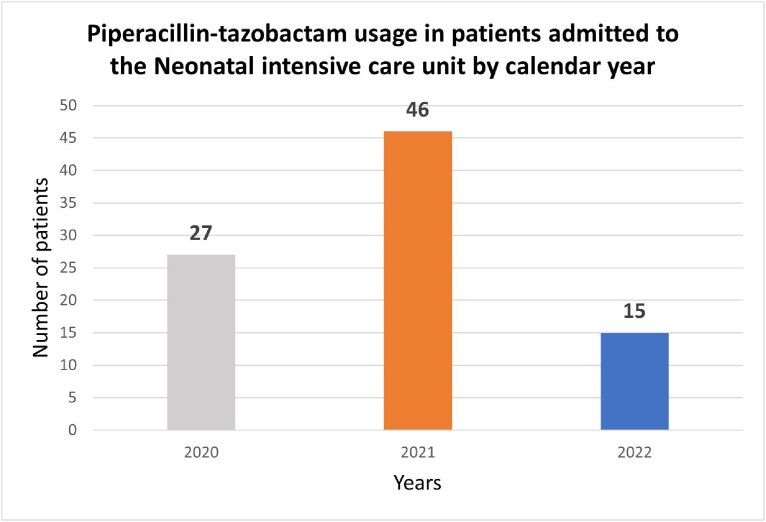
Piperacillin-tazobactam usage in newborns and infants admitted to the Neonatal intensive care unit (NICU).

Piperacillin-tazobactam was administered to a total of 27 patients admitted to the NICU in 2020, 46 patients in 2021, and only 15 patients in 2022. The medication usage evaluation (MUE) was presented by the Pediatric Antimicrobial Stewardship committee in April 2022.

**Conclusion:**

Neonates and premature infants can represent a challenging patient population for Pediatric ASPs’ interventions. Before the introduction of this committee, there was no tracking of the use of broad-spectrum antibiotics in patients admitted to the NICU. We reported a possible overuse of piperacillin tazobactam for early and late onset sepsis, as well as NEC. Our interventions caused a dramatic reduction in the administration of this broad-spectrum antibiotic in the NICU.

**Disclosures:**

**All Authors**: No reported disclosures

